# Non-Aqueous Electrodeposition and Characterization of AlCrCuFeNi High Entropy Alloy Thin Films

**DOI:** 10.3390/ma15176007

**Published:** 2022-08-31

**Authors:** Beatrice-Adriana Serban, Mihai-Tudor Olaru, Ioana-Cristina Badea, Dumitru Mitrica, Marian Burada, Ioana Anasiei, Mihai Ghita, Albert-Ioan Tudor, Cristian-Alexandru Matei, Ana Maria Julieta Popescu, Virgil Constantin, Florina Branzoi, Cristian Dobrescu, Nicolae Constantin

**Affiliations:** 1 National R&D Institute for Non-Ferrous and Rare Metals, 102 Biruinței Blvd., 077145 Pantelimon, Romania; 2 ‘Ilie Murgulescu’ Institute of Physical Chemistry, Romanian Academy, 202 Splaiul Independentei, 060021 Bucharest, Romania; 3 Engineering and Management of Obtaining Metallic Materials Department, University Politehnica of Bucharest, 313 Splaiul Independentei, 060042 Bucharest, Romania

**Keywords:** high-entropy alloys, mathematical modelling, electrodeposition, thin films, corrosion resistance

## Abstract

Materials used in the marine industry are exposed to extreme conditions, so it is necessary to meet remarkable characteristics, such as mechanical resistance, low density, and good corrosion resistance. The challenging environment requires continuous performance improvements, so this work is focused on developing new materials with superior properties, using the electrochemical deposition technique, which are convenient for marine engineering. High-entropy alloys have been attracting tremendous interest in many applications, due to their simple crystal structures and advantageous physical-chemical properties, such as high strength, anti-corrosion, erosion, and electro-magnetic capabilities. To identify the most appropriate compositions, MatCalc software was used to predict the structure and characteristics of the required materials, and thermodynamic and kinetic criteria calculations were performed. The modelling processes generated a series of optimal compositions in the AlCrCuFeNi alloy system, that are suitable to be used in anticorrosive and tribological applications. The composition and morphology of the obtained high entropy alloy thin films revealed a uniform structure, with a small grain profile. The corrosion resistance was investigated in artificial seawater to observe the behavior of the newly developed materials in demanding conditions, and the results showed improved results compared to the copper foil substrate.

## 1. Introduction

**Figure 1:**
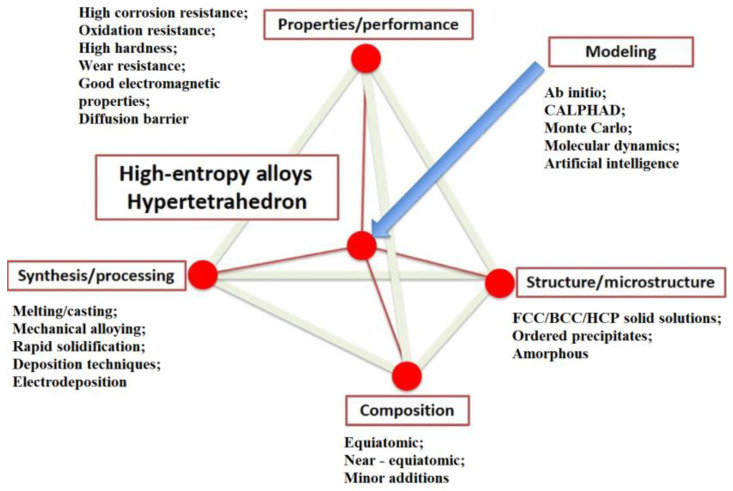
The hypertetrahedron of high entropy alloys [3].

Although the civilizational journey began with the exploration of native metals [1], the discovery of the first alloys and their obtainment techniques brought superior properties and new applications, in different fields of activity [2]. Distinct from the conventional alloys, based on one main element concept, high-entropy alloys are composed of five or more principal elements, so they are considered a new class of metallic materials with a different synthesis strategy [3]. The microstructure and properties of high-entropy alloys [4] are influenced by many factors, including the four core effects: The interference between the high entropy effect and complex phase formation (thermodynamics); the sluggish diffusion effect has the potential of slowing down the phase transformation (kinetics); the severe lattice distortion effect can modify the properties to an extent (structure); and the cocktail effect produces surplus to the amount anticipated by the alloying rule, because of the interactions between atoms and severe lattice distortion (properties) [5,6]. As a consequence of these particularities, high-entropy alloys have the tendency to generate structures based on simple solid solutions (Figure 1). 

Recent research indicated that high-entropy alloys represent a new frontier in metallurgy because of their improved performance [7,8] compared with conventional alloys: Higher corrosion and oxidation resistance, good electromagnetic properties, high hardness and wear resistance, and high strength (Figure 1).

In order to develop new materials with special properties that are suitable for marine engineering [9], it is necessary to identify the optimal compositions of high-entropy alloys. The constituent elements have a major impact on a material’s properties, which should meet the requirements of society by reducing material and energy consumption [10,11]. To improve corrosion resistance and thermal stability, Al, Cu, and Cr are the most used non-ferrous metals; alloying with Fe and Ni produces a malleable material with improved toughness and wear resistance. Moreover, the addition of inexpensive elements will have a positive economic impact [12,13] by developing new materials with reduced manufacturing costs. 

The most appropriate high-entropy alloy compositions were chosen by applying kinetic and thermodynamic criteria. The mixing enthalpy, ΔH_mix_, is an important factor in obtaining intermetallic phases in the alloy structure. Thereby, the criteria of solid solution formation were related to the Hume–Rothery rule, where insignificant dissimilarities in electron valence, electron radius (δ), and electronegativity (Δx) are relevant in the selection process of the alloys. To evaluate the influence of ΔH_mix_ on solid solution formation, the Ω factor was introduced [14]. To predict the formation of sigma phases in high-entropy alloys, it was established that a content exceeding 40% is needed for the sigma-forming coefficient (PSFE) [15,16]. 

High-entropy alloys can be prepared by various methods, of which the most common is the melting–casting process, where induction or arc furnaces are the most used aggregates. Other frequently used synthesis methods are mechanical alloying [17] and rapid solidification [18]. High-entropy-alloy coatings can be obtained using deposition techniques, such as laser cladding [19] and magnetron sputtering [20] (Figure 1) [3]. 

Considering the tendency of decreasing the materials and energy resources that are involved in the obtainment process of high-entropy alloys while increasing efficiency [21], a new manufacturing technique was developed named electrochemical deposition [22]. Furthermore, electrodeposition has other advantages, including a low-temperature process [23], reduced time and costs, and the usage of simple equipment. 

Pavithra C. et al. studied the obtainment of new aqueous-medium-assisted nanocrystalline dual-phase high-entropy alloy thin films containing Co-Cu-Fe-Ni-Zn elements, using the electrochemical deposition technique [24]. The element selection was accomplished based on their ability to be electrochemically reduced in aqueous electrolytes. Difficulties that appear in the high-entropy alloy electrodeposition process in an aqueous medium can be reduced by applying a pulse and engineering other experimental parameters such as pH, applied potential, etc. [25,26]. 

The present work is focused on developing new high-entropy alloys from the AlCrCuFeNi system, using the electrochemical deposition method, with the advanced properties needed in marine engineering [27,28]. To identify the most suitable compositions from the analyzed system, kinetic and thermodynamic criteria were calculated [29], along with the CALPHAD modelling method [30,31]. The resulting samples were physically, chemically, and electrochemically investigated, to identify the properties of HEAs manufactured with this new inexpensive and efficient method.

## 2. Materials and Methods

High-entropy alloys have a complex internal structure, so it is suggested to use modelling instruments to obtain the characteristics needed in marine engineering. To quantify Gibbs free energy in the analyzed system, the modelling process is based on the CALPHAD (Calculation of Phase Diagrams) method. The software used in thermodynamic and kinetic simulation to make the selection process of multicomponent alloys more efficient is MatCalc Pro edition, version 6.03 (Vienna, Austria). Criteria simulation was performed by means of the multi-objective module from MATLAB software (MathWorks, Natick, MA, USA, version R2019a).

The films of the selected high-entropy alloys were obtained by accomplishing electrochemical deposition experiments at room temperature (297 K). To obtain high-entropy alloy films via the potentiostatic electrodeposition technique, a copper foil substrate and organic media were used (Table 1). To apply this method, preceding stages are needed, such as polishing the copper substrate and rinsing it with nitric acid solution and distilled water.

In this work, the composition of a multicomponent alloy with great potential in marine engineering was studied, owing to the superior corrosion resistance. The selected alloys are from the AlCrCuFeNi system, and the deposition characteristics were analyzed by varying the deposition potential. Following these processes, two specimens were obtained, whose parameters are presented in Table 2. For both experiments (D1 and D2), the same electrolyte was used, but dissimilar strategies of applying the deposition potential. The first experiment was performed with variable potential, between −1 and −2.8 V, and a scan rate of 50 mV/s, for 50 cycles (Table 2), and the second experiment was realized on a constant deposition potential of −3 V for 30 min. The thin-film specimens were subsequently annealed at 350 °C for 3 h, in an inert Ar atmosphere (>99.9% purity), using a CARBOLITE CTF 12/100/900 electric furnace (Hope, Hope Valley, Sheffield, UK).

The chemical composition of the alloys was determined by inductively coupled plasma spectrometry (ICP-OES) using an Agilent 725 spectrometer (Santa Clara, CA, USA). The samples were prepared from powders, obtained by scraping the deposited material on the Cu substrate. The deposited films were also analyzed by scanning electron microscopy (SEM) with an FEI Quanta 3D FEG microscope (FEI Europe B.V., Eindhoven, The Netherlands), operating at 20–30 kV.

The tests were performed using a NANOVEA M1 Scratch Tester (Irvine, CA, USA). The pressing force of the 50 µm radius Berkovich-type diamond conical indenter increased continuously from 1…30 N, with a loading rate of 5…15 N/min, over the distance of 5…15 mm, with advance speeds of 2.6…5.2 mm/min. The Micro Stics Nanovea data processing software was used to process the obtained results. 

The corrosion tests were performed with Voltalab 80 PGZ 402, equipment supplied by a special software Volta Master, vers. 7.0.8. The testing process followed previous literature trials [32,33]. The corrosion medium was based on natrium chloride solution 3.5%. The linear polarization curves were made with the electrode potential swept by ±20 mV, versus OCP (open circuit potential). The Tafel curves were drawn based on a polarization experiment at a constant scan rate of 0.166 mV⋅s^−1^, with a shifted potential within ±250 mV vs. E_OCP._

The electrochemical impedance spectroscopy (EIS) techniques were obtained with open-circuit potential in the frequency range of 100 kHz to 40 mHz with an AC wave of ±10 mV (peak-to-peak), and the impedance tests were achieved at a rate of 10 points per decade change in frequency. The Nyquist plots were fitted by ZSympWin software, version 3.6, in order to determine the equivalent circuits. All electrochemical experiments were carried out at room temperature (25 ± 1 °C).

## 3. Results

### 3.1. The Influence of Concentration on the Structure of High-Entropy Alloys

The MatCalc simulation program was used to analyze the redistribution of solid solutions in the solidification operation.

**Figure 2:**
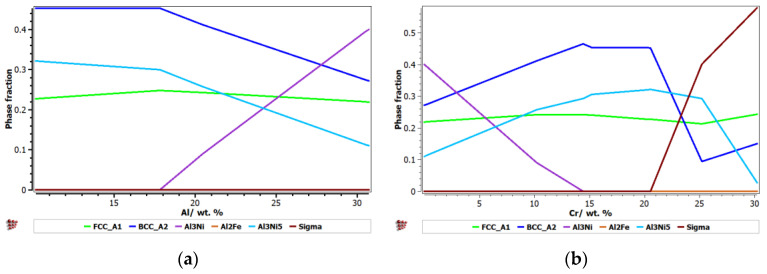

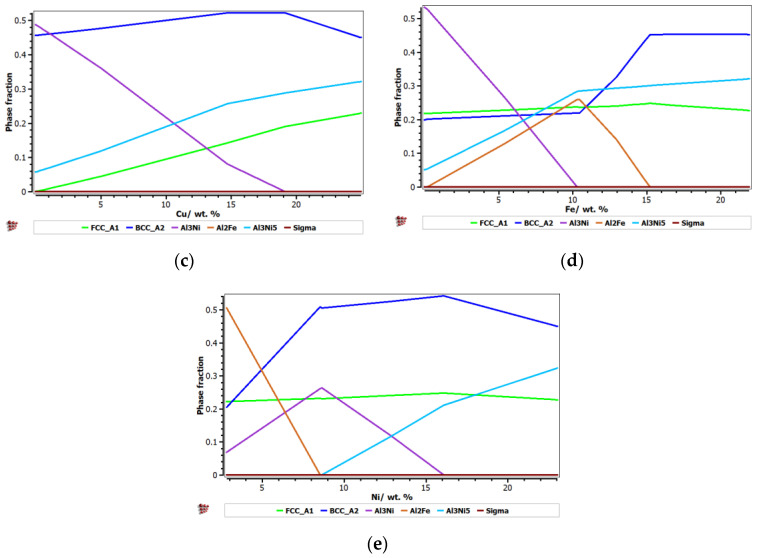
Phase proportions depending on the variation of Al (**a**), Cr (**b**), Cu (**c**), Fe (**d**), and Ni (**e**).

In Figure 2, the evolution of the phases that can be found in the AlCrCuFeNi alloy system is presented, depending on the variation of Al, Cr, Cu, Fe, and Ni.

It can be observed that as the aluminum content increases, the proportion of the BCC–A2 solid solution and Al_3_Ni_5_ intermetallic phase decreases. The addition of 18 wt% Al determines the formation of the Al_7_CuFe phase, which takes over elements from the other phases’ composition, which begin to decrease. It is preferable that this compound would not be present in the alloy, because it would limit the optimal composition of Al on 18 wt%. Low concentrations of Cr favor the formation of the hard and very stable Al_3_Ni compound, and if the percentage of chromium exceeds 20 wt%., a σ brittle phase can appear. Therefore, an optimal chromium content of 15–20 wt% is preferable. Similar to Cr, Cu encourages the formation of the unwanted Al_3_Ni phase, up to approximately 20 wt%. Exceeding 20 wt%. Cu, an equimolar structure can be maintained. The Fe composition is also very important, as is closely related to the formation of the sigma phase. At up to 15 wt%. Fe, a structure with a substantial content of intermetallic phases is found. The minimal three-phase structure occurs at a higher percentage, which is considered optimal. Comparable with other transitional elements, Ni can easily stabilize a large number of compounds at concentrations below 20 wt%. The optimal composition will similarly be in the high-entropy area. 

### 3.2. Multi-Objective Mathematical Optimisation

Optimization of the results was performed using the MATLAB program. The analysis method was multi-objective optimization “gamultiobj” by the Pareto logic genetic method. Thermodynamic and kinetic criteria, represented in the form of objective functions, were taken into account, and the composition of the elements varied from 0 to 1 in mole fraction. The program achieved an optimal possible distribution of the results by analyzing the concentration variation on the chosen domains and boundary conditions. For the proposed alloy system, it is considered that the amount of the molar concentration of the elements should be equal to one, the optimal limit for each criterion should be mentioned, and the variation of the elements’ fraction should be in the range between 0 and 1. By performing the optimization calculation, a high number (18 for the alloy system) of compositions resulted. The optimized alloys have close values of element concentrations, approximately Al_0.1_Cr_0.35_Cu_0.15_Fe_0.12_Ni_0.28_, which was the selected alloy for the experimental trials.

### 3.3. Simulated Phase Diagram of the Selected Alloy Composition

**Figure 3:**
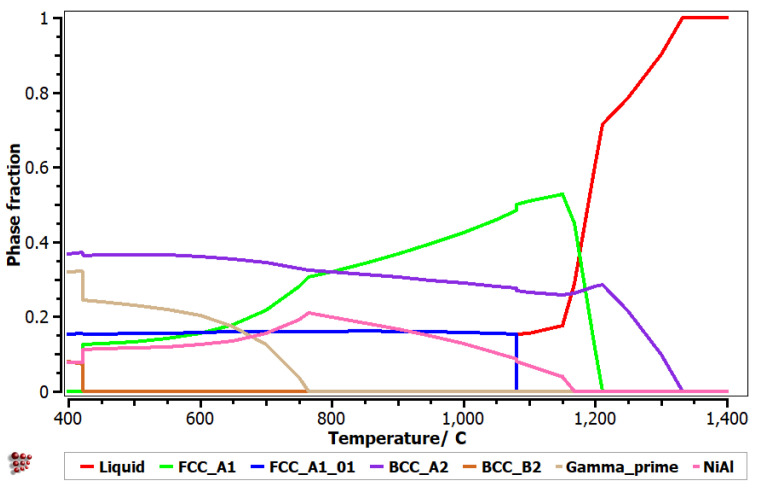
Phase diagram for Al_0.1_Cr_0.35_Cu_0.15_Fe_0.12_Ni_0.28_ alloy.

A simulated phase diagram showing the phase fraction over temperature was provided for the selected alloy composition by means of MatCalc 6.03 software (Figure 3). 

The simulation results for the Al_0.1_Cr_0.35_Cu_0.15_Fe_0.12_Ni_0.28_ alloy indicate a predominant BCC–A2 and FCC–A1 phase structure, with a higher proportion of the FCC phase at high temperatures. Furthermore, the BCC–A2 and NiAl intermetallic phases have higher proportions at room temperature. The gamma prime phase is also met at low temperatures in significant proportions. However, gamma prime represents a softer intermetallic-based structure of the L1_2_ type phase and does not substantially affect the alloy hardness. BCC-B2, which is a known hardening phase, is indicated to be stable only up to 400 °C. Even if the simulation results show a complex structure for the selected alloy, the solid solution formations are predominant. Less brittle behavior is suggested at higher temperatures due to the higher percentage of the FCC phase. The presence of solid solution structures and the increased stability at high temperatures recommend the Al_0.1_Cr_0.35_Cu_0.15_Fe_0.12_Ni_0.28_ alloy for corrosion resistance layers or thermal barriers. 

### 3.4. Experimental Results

#### 3.4.1. Chemical Characterization 

The chemical composition of the samples experimentally obtained and the nominal composition are presented in Table 3. It can be observed that the characterization results for sample D1 are significantly different than the nominal composition, while D2 presents much more similar results. The average difference between experimental and nominal compositions is presented in Table 3 to highlight the observations. Due to the large compositional differences, the D1 experiment was not considered for subsequent heat treatment and microstructural and corrosion characterization. 

#### 3.4.2. Microstructural Characterization

**Figure 4:**
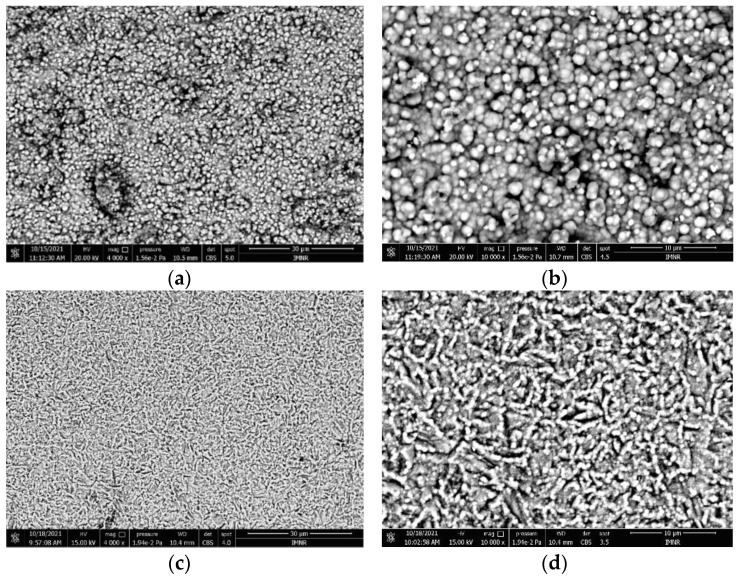
Microstructures of deposited D2 sample (**a**,**b**) under different magnification and heat-treated D2TT sample (**c**,**d**) under different magnification.

**Figure 5:**
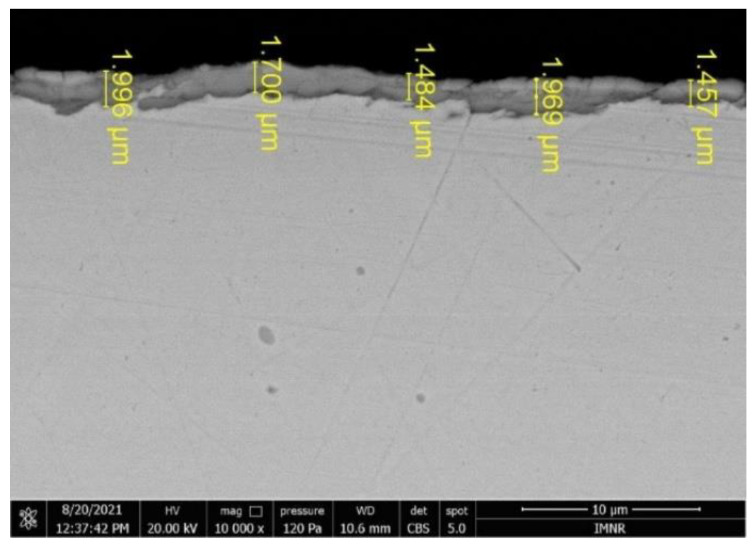
The size of the deposited layer for D2 sample.

SEM analyses revealed the microstructure (Figure 4) and size of the deposited layer (Figure 5), characteristic of the studied alloy in as-deposited (Figure 4a,b) and heat-treated (Figure 4c,d) states. 

**Figure 6:**
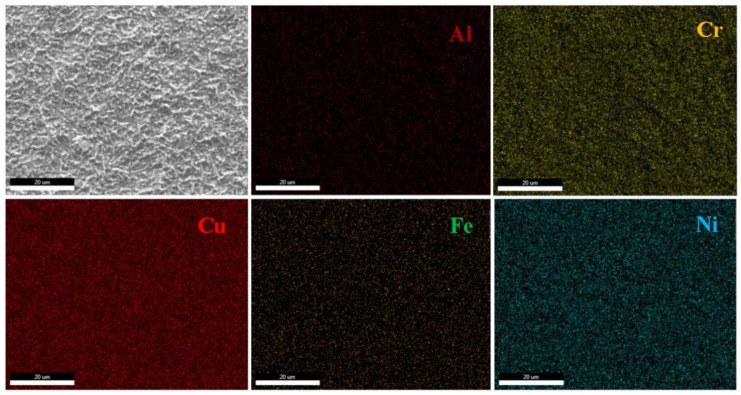
EDS mapping results for the heat-treated alloy film, on the D2 sample.

The deposited samples showed a relatively homogenous structure with fine grains grown from separate nucleation sites, typical for electrochemically deposited thin films. The as-deposited layer presented several areas where the deposition was not homogenous, shown as darker formations in the structure. On the other hand, the heat-treated sample presented a significantly improved structure, with a better distribution on the substrate layer, resulting in a subsequent uniform structure. The grain size of the heat-treated layer is smaller and has an elongated shape. This could be the cause of the formation of alloy phases by elemental diffusion and recombination. There are very small cracks observed in the microstructural analyses, which shows the good adherence of the deposited layer. The transversal section of the specimen shows a continuous and well-defined deposited layer with a thickness varying between 1.5 and 2 µm. Mapping results obtained through SEM-EDS analyses (Figure 6) showed a uniform deposition of the elements on the film surface. The determined composition for Al, Cr, Fe, and Ni was similar to the values obtained by chemical analyses through ICP-OES, after respecting the ratio between them. The value for Cu was considerably higher (51 at.% Cu) and was not taken into consideration, because Cu is also the film substrate and is identified by EDS analyses in the final spectrum. 

**Figure 7:**
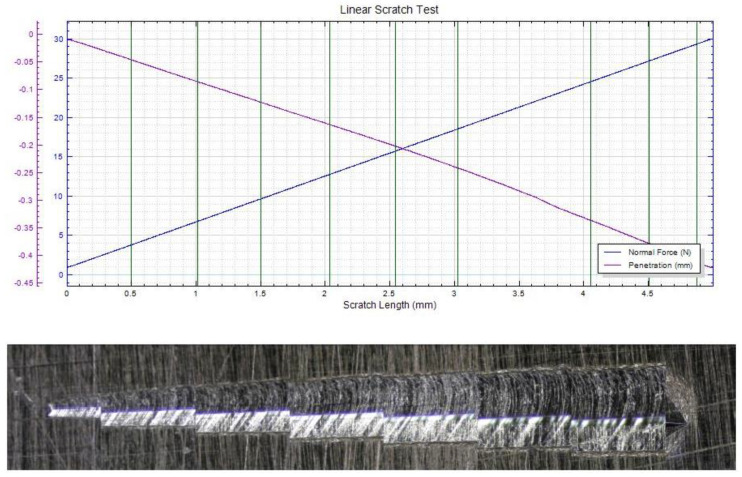
Scratch test result of the Cu substrate (pressing force: 1…30 N, force rate 15 N/min, length: 5 mm).

**Figure 8:**
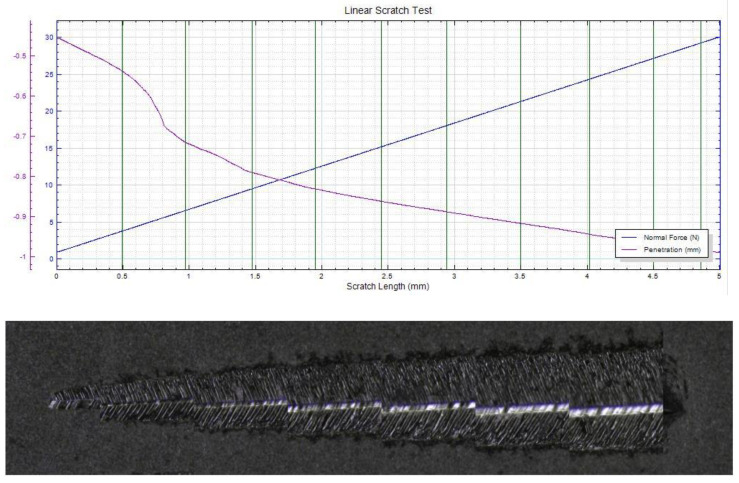
Scratch test result of D2 sample (pressing force: 1…30 N, force rate 10 N/min, length: 5 mm).

**Figure 9:**
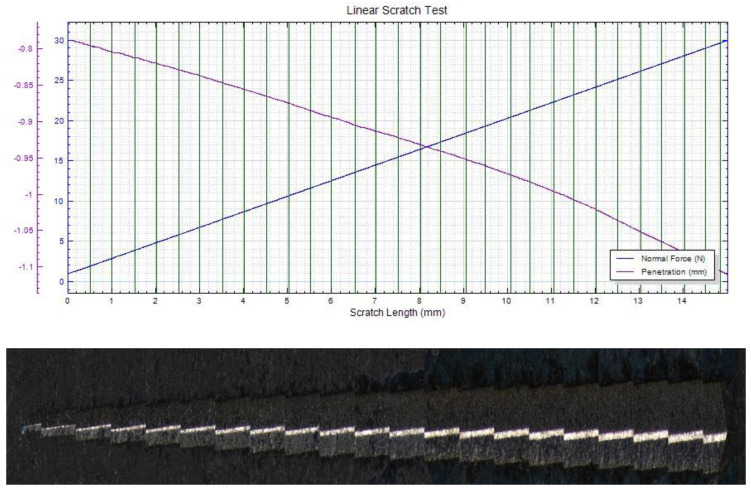
Scratch test result of D2TT sample (pressing force: 1…30 N, force rate 10 N/min, length: 15 mm).

In Figure 7, Figure 8 and Figure 9, the scratch test results for copper substrate and the obtained D2 samples are presented, before and after the heat treatment. 

**Figure 10:**
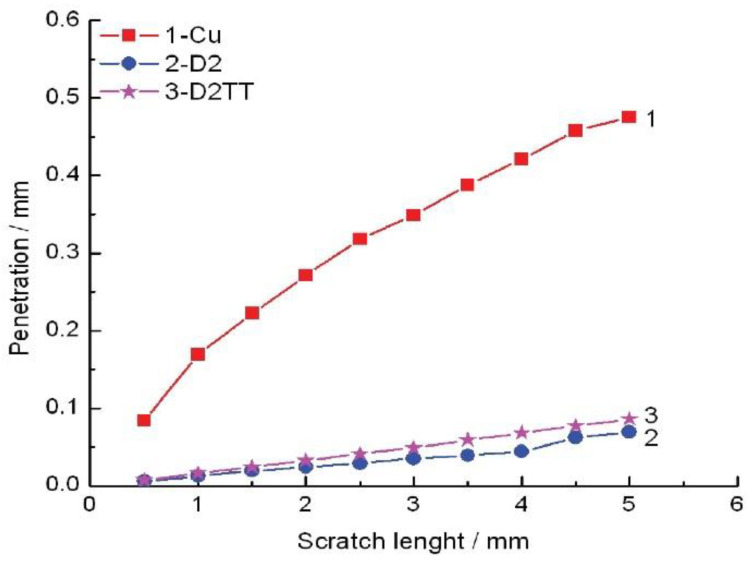
Comparative analysis between the indenter penetration depth penetration for Cu, D2, and D2TT specimens.

The mechanical “scratch” tests of adhesion of HEA films, performed with pressure forces of 1…30 N, a loading speed of 5…15 N/min, an advance speed of 2.6…5.2 mm /min, and a distance of 5…15 mm, showed that the deposits obtained are adherent. The thermally treated film shows a uniform behavior, with a higher scratch resistance compared to the untreated film and the Cu substrate. In Figure 10, a comparative analysis of the indenter penetration depth variation of three samples (copper substrate, D2, and D2TT) is presented, in order to easily observe the superior mechanical properties of D2 and D2TT specimens. The obtained samples show a high degree of homogeneity of the deposited layers, better adhesion, and a lower degree of exfoliation than the Cu substrate. 

### 3.5. Corrosion Tests

The purpose of this study is to examine the corrosion protection properties of the thin films deposited on the copper substrate and their behavior in an aggressive environment and especially in a NaCl solution. The inhibitory performance of these films was evaluated by potentiodynamic polarization curves and electrochemical impedance spectroscopy (EIS). The results were presented against the Cu substrate’s performance.

#### 3.5.1. Potentiodynamic Polarization Studies 

**Figure 11:**
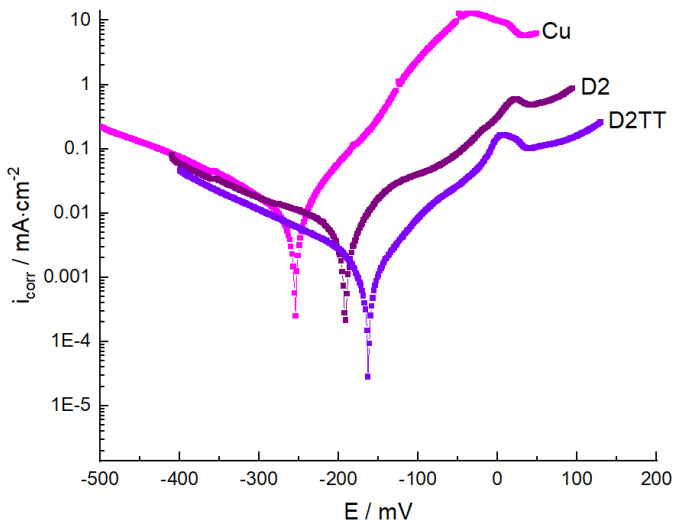
The polarization curves of electrodes covered and not covered with protective films.

One of the best methods of protection against the corrosion of metal electrodes in an aggressive environment is the electrodeposition of thin protective films, which influence both the anodic reaction and the cathodic reaction. The polarization behavior of the as-deposited and heat-treated layers in corrosion tests in a 3% NaCl solution at room temperature is presented in Figure 11. From the analysis of Figure 11, it can be seen that both the cathodic and anodic polarization curves indicated a lower current density in the presence of protective films. The polarization curves showed a positive shift of the corrosion potential for coated electrodes, which indicates better protection of the electrode surface against corrosion by the deposited films. This behavior demonstrates that the protective films had a significant effect on the cathodic and anodic reactions of the electrochemical process. From the analysis of the Tafel curves presented in Figure 11 and Table 4, where the measured and calculated corrosion kinetic parameters are presented (open circuit potential (OCP), polarization resistance, corrosion potential (E_corr_), corrosion current density (i_corr_), and corrosion rate), the differences between the coated and non-coated samples can be identified.

From the comparative analysis of samples D2 and D2TT in relation to the uncoated sample and the analysis of polarization curves with corrosion kinetic parameters, it can be seen that heat treatment improved the corrosion resistance of the alloy. This analysis of the polarization curves indicated that the protective films of the electrodes had an important influence on the cathodic and anodic reactions of the electrochemical process, and it was noted that the electrodeposited film prevented the attack of corrosive ions (Cl^−^) on the electrode surface. Furthermore, from this analysis, one can observe that the coated electrodes had better protective properties with an efficiency of 50% in relation to the uncovered electrodes.

#### 3.5.2. EIS Analysis

The corrosion behavior, the corrosion mechanisms, and the adsorption phenomena of the films deposited on the surface of the electrodes in a 3% NaCl medium were studied by electrochemical impedance spectroscopy (EIS). EIS measurements were performed over a frequency range of 100 kHz–40 mHz with an open circuit potential (OCP) and a sinusoidal AC voltage waveform of ±10 mV (peak to peak). In Table 5, the electrochemical parameters used to effectuate EIS analyses are presented. The experimental results presented the electrochemical properties of the protective films at the electrode–electrolyte interface. 

**Figure 12:**
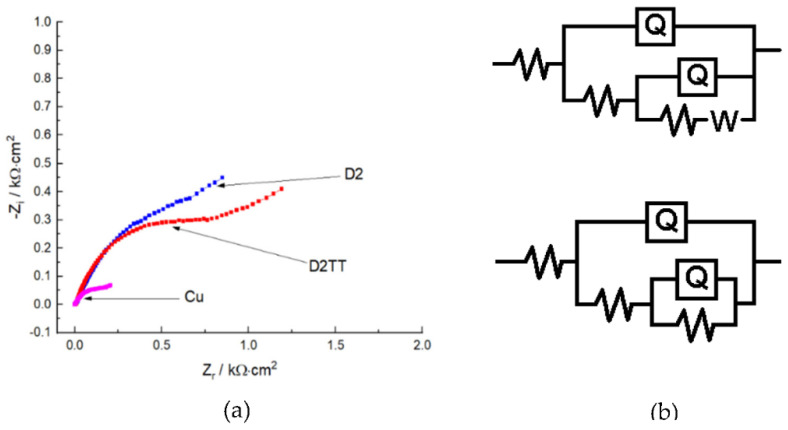
Nyquist diagrams of deposited protective films (**a**) and equivalent circuit representing the Nyquist diagram (**b**).

The Nyquist diagrams for the electrode covered with protective films and not covered in the 3% NaCl solution are presented in Figure 12.

Examining Figure 12a, it can be seen that the impedance diagram of the uncovered electrode can be described by two semicircles, a smaller one at a high frequency followed by a larger one at medium and low frequencies. The first semicircle is attributed to the formation of the corrosion product film and is represented by the corrosion film resistance and the corrosion film capacity. The second semicircle is attributed to the diffusion of copper cations (soluble CuCl^2−^) in the solution and is characterized by the charge transfer resistance and double-layer capacitance and Warburg impedance (W). The presence of Warburg impedance indicates the diffusion of soluble species. Corrosion of the bare electrode is under mixed control: By the dissolution of copper and diffusion of copper cations within the electrolyte. The Warburg impedance takes into account the process of diffusion of soluble copper compounds from the surface of the electrode into the volume (bulk) of the solution. The Nyquist impedance plot of the bare electrode is a characteristic of a dissolution process with the precipitation of a film of corrosion products on the surface of the electrode. 

The Nyquist diagram of electrodes covered with protective films is modeled using the equivalent circuit shown in Figure 12b. Moreover, the parameter values had a very good fit in the impedance diagrams and are significantly more distinctive compared to those obtained for the uncovered electrode. In the case of coating/protection films, in the high-frequency range. the first capacitive loop was attributed to the characteristics of the coating film/electrolyte interface and is represented by the coating resistance and the coating capacitance. The second half-circle in the medium- and low-frequency range is attributed to the interface of the coating/Cu film and the processes occurring under the deposited film, which can be characterized by the resistance to charge transfer and the capacity of the double layer.

From the analysis of the Nyquist diagram, it can be appreciated that the response to the impedance of the coated electrode was significantly modified by the deposition of the protective film, which implies that the presence of the protective film was accentuated. It can be deduced that the electrochemical impedance loop diameter for the coating film was larger than that for the uncoated sample, indicating a higher protection efficiency for the coated electrode.

The EIS data were analyzed, and the use of the equivalent electric circuit shown in Figure 12 was proposed. An equivalent electrical circuit for Cu in NaCl has been proposed to match the impedance spectra of bare copper, and the component parameters are electrolyte resistance (Rs); the phase element constant, which is related to the film capacitance of corrosion CPE_f_ (Q_1_-Yo); Rf represents the resistance of the corrosion film; CPE_p_ (Q_2_-Yo) is the constant of the phase element that is connected to the corrosion compounds, namely the capacitance of the double layer; R_ct_ represents the load transfer resistance; and W the Warburg impedance. For the coated electrodes (D2, D2TT), an equivalent circuit is suggested to match the impedance spectra of the coating film, where Rs is the resistance of the solution, CPEc (CPE1) is the constant of the phase element and is related to the coating capacitance, R_c_ represents the resistance of the coating, CPE_ct_ (CPE2) is the constant of the phase element that represents all the frequencies dependent on the electrochemical processes, and namely, the double layer capacitance, and R_ct_ represents the charge transfer resistance.

Therefore, in the studied frequency range, an equivalent circuit model was proposed (Figure 12), resulting from fitting and exploring the EIS experimental data. In this case, the phase element constant CPE is introduced into the circuit instead of a pure double-layer capacitor (C_dl_) to provide a more accurate fit: C_dl_ = Y_0_ (ω_max_)^n−1^; CPE is used to describe the deformation of the semicircle capacitive, which corresponds to the heterogeneity of the surface due to the presence of roughness and impurities on the electrode surface. The CPE impedance can be defined as Z_CPE_ = Y_0_^−1^ (jω)^–n^ where ω is the angular frequency (ω = 2πf), j is the imaginary number (j_2_ = −1), Y_0_ is the amplitude compared to capacitance, and n is the change of phase. The value of n gives details on the degree of inhomogeneity of the metal surface. A higher value of n is associated with a lower surface roughness (reduced inhomogeneity). The constant of the phase element can be considered resistance (when n = 0, Y_0_ = R), it is considered capacitance when (n = 1, Y_0_ = C), inductance when n = −1, Y_0_ =1/L, or Warburg impedance when n = 0.5, Y_0_ = W, depending on the value of n. It can be said that higher values of R_ct_ for the coating films are attributed to the barrier effect with high efficiency, and lower values of CPE for the coating films provide greater corrosion protection of the coated electrode studied in a corrosive environment. It is very obvious that a very good fit was achieved with this equivalent circuit model, which is in good agreement with the data obtained from the experimental potentiodynamic bias measurements, as can be seen for all the experimental impedance data.

**Figure 13:**
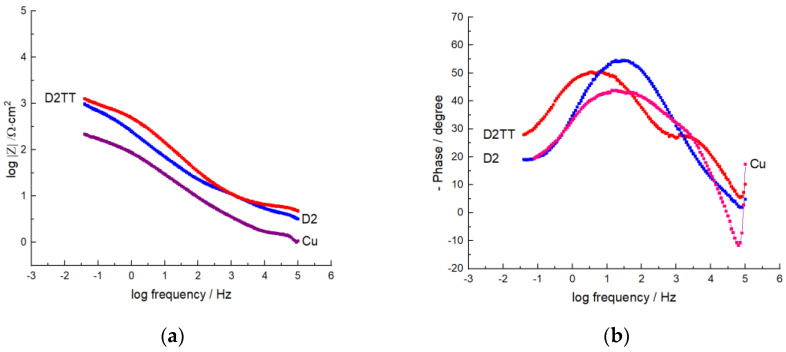
Bode diagrams with impedance (**a**) and frequency (**b**) curves, of the deposited protective films.

Furthermore, higher values of R_ct_ and lower values of C_dl_ denote the best corrosion protection performance of the electrode covered with protective films in a sodium chloride environment. From the analysis of the EIS data, it can be concluded that coated electrodes had much better polarization behavior and provided excellent protection compared to uncovered electrodes. The parameters obtained by analyzing the equivalent circuit of covered and uncovered electrodes are presented in Table 5. The Bode plots shown in Figure 13a,b are consistent with the Nyquist plot, and it can be seen that a higher value of the impedance modulus (Z_mod_) at low frequency values describes better surface corrosion protection efficiency, which was observed for the covered electrodes.

The Nyquist and Bode plots suggest that the protective film on the electrode surface stopped the corrosion process and acted as a barrier through the charge transfer phenomenon. Moreover, the EIS data are in good agreement with the results obtained by potentiodynamic polarization.

## 4. Conclusions

This paper presents the selection, obtainment, and characterization processes of high-entropy alloys. An AlCrCuFeNi alloy system was discussed to develop new HEA coatings with good corrosion resistance that are suitable for marine engineering. The alloy films were prepared by the potentiostatic electrodeposition technique, using a copper foil substrate and organic media. 

The alloy selection was performed by means of modeling software, using current criteria calculations and the thermodynamic database, and the optimization of the results was performed using the MATLAB program. The selected Al_0.1_Cr_0.35_Cu_0.15_Fe_0.12_Ni_0.28_ alloy presents a mixed BCC–A2 and FCC–A1 phase structure, with a higher proportion of FCC phase, at high temperatures. In addition, gamma prime was found with significant proportions at lower temperatures. Based on the structural characteristics, the Al_0.1_Cr_0.35_Cu_0.15_Fe_0.12_Ni_0.28_ alloy contains a large number of solid solution phases with high stability at higher temperatures, encouraging their consideration for applications that require corrosion resistance or thermal barrier protection. 

SEM analyses of the deposited layers of the selected alloy revealed a relatively uniform structure with a small grain profile. The heat-treated sample showed a more compact surface structure, with a smaller grain size. There are no obvious cracks in the deposited layers, which suggests good adherence to the deposited layer. 

The adhesion behavior of the deposited samples showed improved values even against the compact Cu substrate sample. The thermally treated film shows a uniform scratch profile compared to the untreated film. Both samples have better scratch resistance than the Cu substrate. The analyzed specimens showed a high degree of homogeneity of the deposited layers, better adhesion, and a lower degree of exfoliation.

The corrosion resistance of the obtained materials was tested in 3.5% NaCl solution by the potentiodynamic polarization and electrochemical impedance spectroscopy methods. The results demonstrated that high-entropy alloy films showed superior results compared with the copper foil substrate. The heat-treated HEA film offers supplementary protection in demanding conditions and has promising results for further investigations. 

## Figures and Tables

**Table 1:** Electrolyte chemical composition.

Material	AlCl_3_ mol/L	CrCl_3_ × 6H_2_O mol/L	CuCl_2_ mol/L	FeCl_2_ × 4H_2_O mol/L	NiCl_2_ × 6H_2_O mol/L	LiCl_0.4_ mol/L
Electrolyte	0.02	0.07	0.03	0.02	0.05	0.1

**Table 2:** Deposition parameters for the two specimens.

Alloy	Electrolyte Type	Potential, V	Time, Min	Scan Rate, mV/s
D1	El	−1.0 to −2.8 V	-	50
D2	El	−3.0 V	30 min	-

**Table 3:** Chemical composition of the Al_0.1_Cr_0.35_Cu_0.15_Fe_0.12_Ni_0.28_ alloy expressed in weight percentage.

State	Al	Cr	Cu	Fe	Ni	Average Difference
nominal	5.03	33.98	17.8	12.51	30.68	-
D1	14.94	4.72	53.20	21.63	5.51	21.772
D2	9.6	31.5	15.4	12.9	30.6	1.984

**Table 4:** Corrosion kinetic parameters for the studied samples.

Sample	E_corr_, _(mV)_	I_corr_, _µA/cm_^2^	R_p_, _ohm·cm_^2^	ba	bc	R, _mpy_	P, _mm/an_	E, _%_
Cu	−263	7.73	1782	63	−125	3.66	0.093	-
D2	−192	5.23	3400	78	−185	2.531	0.064	34
D2TT	−205	4.01	2800	93	−96	2.012	0.051	50

**Table 5:** Electrochemical parameters for EIS analyses.

System	Rs	Q_1_-Yo	Q_1_-n	Rf/Rc	Q_2_-Yo	Q_2_-n	R_ct_	W	χ
Cu	16.9	1.143 × 10^−4^	0.705	232	1.642 × 10^−4^	0.622	1843	0.002246	6.17 × 10^−4^
D2TT	5.69	0.000402	0.675	572	0.005279	0.79	1315	-	1.26 × 10^−3^
D2	3.73	8.173 × 10^−5^	0.767	9973	0.001029	0.625	1135	0.00603	7.61 × 10^−4^

Rs—solution resistance; Rf—corrosion film resistance for Cu; Rc—the resistance of the coating (for D2, D2TT); Q_1_—the phase element constant related to the corrosion film capacitance; R_ct_—the charge transfer resistance; Q_2_—the capacity of the double layer; W—Warburg impedance.

## Data Availability

Not applicable.
